# Sleep disturbance as a mediator of the relationship between perceived stress and demoralization in hemodialysis patients: a structural equation modeling analysis

**DOI:** 10.3389/fpsyt.2025.1420630

**Published:** 2025-06-13

**Authors:** Yansheng Ye, Jing Zhang, Yane Sun, Jianqing Xu, Qian Xu, Chengrong Song

**Affiliations:** The Sixth Affiliated Hospital of Kunming Medical University/The People’s Hospital of Yuxi City, Yuxi, Yunnan, China

**Keywords:** chronic kidney disease, perceived stress, sleep disturbance, demoralization, hemodialysis patients, mediating role

## Abstract

**Background:**

Demoralization describes a state of existential distress, isolation, impotence, hopelessness, helplessness, and loss of purpose and meaning in life. Demoralization is associated with suicidal thoughts, which could lead to a desire for hastened death. Perceived stress could be viewed as the sense of imbalance between the stressors experienced by an individual in daily life and his or her coping capability. Many studies have tested the relationships between perceived stress, sleep disturbance, and demoralization; however, the mechanism of sleep disturbance has not been fully evaluated.

**Objective:**

To verify the relationship between perceived stress and demoralization and explore the mediating effect of sleep disturbance on the relationship between perceived stress and demoralization in hemodialysis patients.

**Materials and methods:**

A cross-sectional questionnaire survey using convenience sampling from July to August 2022, 547 hemodialysis patients from ten hospitals filled out the Perceived Stress Scale (PSS), Pittsburgh Sleep Quality Index (PSQI), Demoralization Scale (DS), and general information questionnaire. The data were analyzed using SPSS 26.0, and path analysis and structural equation modeling were used to explore the relationships among perceived stress, sleep disturbance, and demoralization.

**Results:**

Perceived stress was significantly and positively associated with demoralization (*P* < 0.01) among hemodialysis patients. Sleep disturbance partially mediated the relationship between perceived stress and demoralization (B = 0.154, 95% CI: 0.102~0.213); the proportion of mediation was 43.75%.

**Conclusion:**

Perceived stress affects demoralization among hemodialysis patients, and sleep disturbance is a mediator in the relationship. Perceived stress in hemodialysis patients should be measured and effectively managed to improve positive effects on demoralization. It is necessary for medical staff to consider implementing perceived stress interventions with an emphasis on construction of sleep strategies to assist hemodialysis patients improve their demoralization.

## Introduction

Chronic kidney disease (CKD) is an emerging global health care epidemic with an increasing prevalence, which has been reported as 11.0% ~ 13.4% worldwide (1). When patients with CKD progress to end-stage renal disease, they need kidney replacement therapy, including kidney transplantation and renal dialysis, to maintain life. China has become one of the most common and prevalent users of maintenance hemodialysis (MHD) therapy in the world, and more than 713,000 people are currently on hemodialysis therapy (2, 3).

Demoralization describes a state of existential distress, isolation, impotence, hopelessness, helplessness, and loss of purpose and meaning in life (4). Demoralization is associated with suicidal thoughts, which could lead to a desire for hastened death (5). An overall prevalence of 17.2% ~ 85.5% for demoralization morbidity has been notified in kidney transplant recipients in Italy (6, 7). Zhuang et al. (8), through an investigation of 278 dialysis patients, indicated that anxiety and depression could significantly predict the level of demoralization (P < 0.01) (8). So far, there has been little research on the relationship between perceived stress, sleep disturbance, and demoralization in hemodialysis patients.

The core feature of demoralization is the sense of impotence caused by uncertainty about the future, whereas the characteristic feature of depression is an overall lack of motivation (9, 10). In addition, there are differences in the treatment of demoralization and depression. The best treatment for demoralization is psychotherapy, such as dignity therapy, whereas severe depression should be treated with medication (9, 10).

Several studies have shown that stress is linked to demoralization. For instance, stress status could significantly positively predict the level of demoralization among cardiac transplant recipients (11). Casu et al. (12) indicated that hospital nurses with increased emotional job stress were more likely to report higher levels of distress and demoralization (12). De Figueiredo et al. (13), through an investigation of 95 outpatients with Parkinson’s disease, found that perceived stress was positively correlated with demoralization (P < 0.01) and could significantly predict the level of demoralization (P < 0.01) (13). Similarly, Wu et al. (14) investigated 99 cardiac transplant recipients and found that stress status was positively correlated with demoralization (P < 0.01) (14). Hemodialysis patients experience many stress, such as economic hardship, symptom burden, and fear of disease progression (14). In Yunnan Province, China, a multiethnic area, very little is known about the association between perceived stress and demoralization among hemodialysis patients.

Many researchers agree that stress is related to sleep disturbance. For example, Liu et al. (15) conducted a study on 1471 adults enrolled in a questionnaire survey and found that perceived stress was positively correlated with sleep disturbance (P < 0.01) (15). Benham (16) found significant positive correlations between psychological stress and insomnia (16). Similarly, Furuichi et al. (17) investigated 2899 adult office workers and found that job stressors were positively correlated with sleep disturbance (P < 0.01) and could significantly predict the level of sleep disturbance (P < 0.01) (17). The study indicated that work stress was significantly correlated with increased levels of insomnia symptoms and that higher work stress led to more serious insomnia symptoms for 3706 participants with jobs in the Swedish longitudinal occupational health survey (18). Despite all these studies, few studies have investigated the relationship between perceived stress and sleep disturbance among hemodialysis patients.

In light of quantitative and qualitative studies, sleep disturbance is linked to demoralization. For instance, Clarke et al. (19), who conducted 30-min interviews with 49 hospitalized patients aged 21 and 83 years with medical illnesses, found that ‘not being able to sleep’ due to discomfort or pain related to their illness was one of the described domains related to demoralization. One study found that early insomnia was positively correlated with demoralization (P < 0.05) in a sample of 288 patients with acute coronary syndrome, meaning that the earlier insomnia occurred, the greater the level of demoralization was (20). Similarly, Chang et al. (21) investigated 121 breast cancer patients and found that demoralization was significantly associated with sleep disturbance, including daytime dysfunction, sleep latency, and subjective sleep quality, but sleep disturbance was not a significant predictor of demoralization in regression analyses. The prevalence of insomnia was 63.73% in hemodialysis patients, and insomnia is one of the most commonly reported symptoms among them (22). The above contradictory results about the relationship between sleep disturbance and demoralization are worth empirically clarifying to establish more effective methods for targeted interventions for hemodialysis patients.

**Figure 1:**
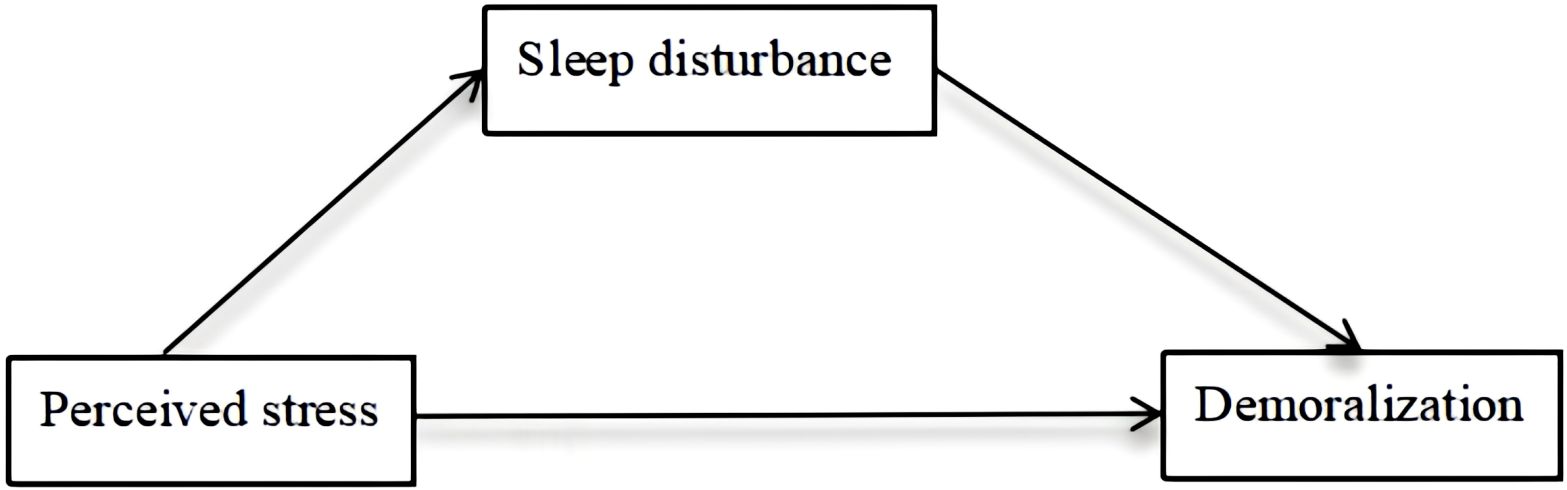
Hypothesized theoretical model.

There is increasing evidence of the relationship among perceived stress, sleep disturbance, and demoralization; however, the mechanism of sleep disturbance has not been fully evaluated. Sleep disturbance, as a mediator variable, was described in self-regulatory resource theory and the three previous studies (23–26). For instance, Ben et al. (24) investigated 579 smokers and nonsmokers and found sleep disturbance as a mediator between early life adversities and depression. Similarly, Guo et al. (26) found that sleep disturbance significantly mediated the relationship between problematic internet use and suicidal behavior in a sample of 20,895 students. The model with the assumption in this study is shown below (**Figure 1**). According to the model, we explored the relationships among perceived stress, sleep disturbance, and demoralization, with the results showing that perceived stress influences demoralization though its effects on sleep disturbance. However, the impact of sleep disturbance on enhancing the relationship between perceived stress and demoralization has received little attention, and few studies have explored the relationships and mechanisms among the three variables in hemodialysis patients.

Based on the research findings reported above, the purpose of this study was (1) to verify the relationship between perceived stress and demoralization in hemodialysis patients and (2) to explore the mediating effect of sleep disturbance on the relationship between perceived stress and demoralization.

## Materials and methods

### Study design and setting

A cross-sectional study was conducted in hemodialysis patients in Yuxi, China, from July to August 2022. The participants were enrolled from ten hospitals (one third-level general hospital and nine second-level general hospitals). The questionnaire pertaining to demographic information, disease-related characteristics, perceived stress, sleep disturbance, and demoralization, which took 10~25 min to complete, was distributed to the hemodialysis patients.

### Participants

Patients were included if they (1) were aged 18 years or older, (2) had received hemodialysis for over 3 months, (3) had received hemodialysis 2~3 times per week, and (4) agreed to participate in this study. Participants were excluded if they met any of the following criteria: (1) Inability to communicate or write normally; (2) A history of psychiatric disorders; (3) Active infectious diseases (e.g., hepatitis B, syphilis, or HIV).

Sample size calculation should consider the general rule of thumb that more than 200 participants should generally be acquired for structural equation modeling (SEM) (27). The inclusion of 547 hemodialysis patients was consistent with the above rule.

### Ethics statement

This study was approved by the Committee of Medical Ethics of the Sixth Affiliated Hospital of Kunming Medical University (No. 2020 kmykdx6937). According to ethical principles, we obtained written informed consent from the hemodialysis patients, and all of them willingly enrolled in this study.

### Measures

#### Measuring tools and contents

Measuring Tools and Contents are follows (**Table 1**).

**Table 1:** Tools and measurements.

Measuring Tools	Measurement contents
The general information form	Gender, age, education level, employment status, income status, marital status, religious belief, the number of complications, dialysis modality, duration of dialysis, primary kidney disease, and history of kidney transplantation
The Perceived Stress Scale	Perceived stress
The Pittsburgh Sleep Quality Index	Sleep disturbance
The Demoralization Scale	Demoralization

#### The general information form

The form had two sections: demographic variables (gender, age, education level, employment status, income status, marital status, and religious belief) and clinical characteristics (the number of complications, dialysis modality, duration of dialysis, primary kidney disease, and history of kidney transplantation). Clinical characteristics were recorded from the participants’ medical records by the researcher. Religious belief was grouped into two categories, i.e., “yes” and “no”. History of kidney transplantation was divided into “yes” and “no”. The categories of other indicators are shown in **Table 2**.

#### Measurement of perceived stress

The Perceived Stress Scale (PSS) was first constructed by Cohen et al. (28) to measure the perceived stress levels of subjects. The Chinese version of the PSS constructed by Yang (29) includes 14 items and two subscales: sense of uncontrol (seven items) and sense of nervous (seven items). Each item is rated on a five-point scale ranging from 0 (never) to 4 (very often). PSS scoring uses two subscales, and the total score ranges from 0 to 56, with a higher score indicating a greater perceived stress level. The PSS is a self-rated scale. The Chinese version of PSS has been validated as an instrument with good reliability for assessing stress levels in Chinese hemodialysis patients (30). The Cronbach’s alpha coefficient was 0.856 in this study.

#### Measurement of sleep disturbance

The Pittsburgh Sleep Quality Index (PSQI) was first constructed by Buysse et al. (31) to measure the sleep disturbance levels of subjects. The structure and scoring system of the Chinese version of the PSQI were the same as those for the PSQI developed by Buysse et al. (32). This scale includes 19 items and seven components: daytime dysfunction, sleep disturbances, subjective sleep quality, sleep duration, sleep latency, habitual sleep efficiency, and the use of sleep medication. Each component score ranges from 0 to 3. PSQI scoring uses seven component subscales, and the total score ranges from 0 to 21, with a higher score indicating poorer sleep quality. The PSQI is a self-rated scale. The Cronbach’s alpha coefficient was 0.817 in the present study.

#### Measurement of demoralization

The Demoralization Scale (DS) was first constructed by Kissane et al. (33) to measure the demoralization levels of subjects. The Chinese version of the DS constructed by Hung (34) includes 24 items and five subscales: helplessness (four items), sense of failure (four items), dysphoria (five items), disheartenment (six items), and loss of meaning (five items). Each item is rated on a five-point scale ranging from 0 (complete disagreement) to 4 (complete agreement). DS scoring includes five subscales, with the total score ranging from 0 to 96; a cutoff point of 30 and above indicates a high demoralization level. The DS is a self-rated scale. The Cronbach’s alpha coefficient was 0.892 in this study.

### Data analysis

#### Primary analysis

The data for perceived stress, sleep disturbance, and demoralization showed normal distributions (tested by the skewness and kurtosis test with SPSS version 26.0). The group differences in demoralization were tested by one-way ANOVA or t tests. The correlations among perceived stress, sleep disturbance, and demoralization were analyzed using Pearson correlation analysis. All statistical tests were two-tailed, with *P* < 0.05 judged as statistically significant.

#### Hierarchical multiple regression analyses

Hierarchical multiple regression (HMR) analyses were carried out to preliminarily test the predictors of demoralization and confirm the mediating effect of sleep disturbance on the relationship between perceived stress and demoralization. Demoralization was considered an outcome variable, and the independent variables (i.e., sociodemographic and clinical characteristics, perceived stress, and sleep disturbance) were divided into 3 steps as follows: Step 1 (Model 1): sociodemographic and clinical characteristics; Step 2 (Model 2): perceived stress; and Step 3 (Model 3): sleep disturbance. If the regression coefficient of perceived stress to the demoralization was significantly reduced from Step 2 to Step 3, it had a partially mediating effect. If the regression coefficient of perceived stress to demoralization was not significantly different (*P* > 0.05), sleep disturbance had a complete mediating effect. The analyses were conducted in stages by successively entering blocks of independent variables in the model.

#### Structural equation modeling analyses

Hayes’ PROCESS macro is a popular statistical tool for analyzing mediation (indirect effects), moderation (interaction effects), and their combination (conditional process models). It uses regression-based path analysis with bootstrap confidence intervals to test hypotheses without normality assumptions.

The potential mediating effect of sleep disturbance was further confirmed using the SPSS PROCESS macro model 4 with 5,000 bootstrap samples, under the bias-corrected 95% confidence interval (CI). Path coefficients (c, a, b, and c’) in the hypothesized model were obtained after analysis. If the 95% CI of the indirect effect did not include zero, the mediating effect was judged to be statistically significant.

## Results

### Hemodialysis patients’ sociodemographic and clinical characteristics

The sociodemographic and clinical characteristics of the hemodialysis patients are shown in **Table 2**. Of the 547 patients, 62.3% were males and 37.7% were females. 31.3% fell within the 18–45 years old and 78.98% lived with spouses. 16.09% were employed. 41.32% had a monthly income of less than 5,000 yuan and 42.96% had an education level of junior school or below. 85.74% lived with family, 9.14% had a religious affiliation, and 90.49% had one or more complications. 23.77% used hemodialysis as a dialysis modality, 10.05% had a dialysis duration of less than one year, and 39.30% with primary kidney disease had primary glomerular disease. There was no significant difference in the level of demoralization among hemodialysis patients by gender, age, marital status, cohabitation status, religious belief, dialysis modality, duration of dialysis, or primary kidney disease. However, there were significant differences in demoralization levels by employee status, monthly income, education level, and complications. The differences in the levels of demoralization among hemodialysis patients with different sociodemographic and clinical characteristics are shown in **Table 2**.

**Table 2:** Sociodemographic and clinical characteristics, and differences of demoralization level by characteristics (N = 547).

Variable	Categories	N (%)	Demoralization (Mean ± SD)
Gender	Male	341 (62.3)	2.85±0.61
	Female	206 (37.7)	2.77±0.69
Age (years)	18-45	171 (31.3)	2.81±0.62
	45-76	376 (68.7)	2.80±0.67
Marital status	Spouses living	432 (78.98)	2.82±0.61
	Others	115 (21.02)	2.76±0.79
Employee status	Yes	88 (16.09)	1.71±0.37
	No	459 (83.91)	3.01±0.46^**^
Monthly income, Yuan	<5,000	310 (56.67)	3.39±0.25
	5,000-10,000	165 (30.17)	2.74±0.20
	≥10,000	72 (13.16)	1.85±0.35^**^
Education level	Junior school and below	235 (42.96)	3.38±0.26
	Senior high school	201 (36.75)	2.69±0.22
	College and above	111 (20.29)	1.79±0.34^**^
Cohabitation status	Lives with family	469 (85.74)	2.79±0.64
	Alone	78 (14.26)	2.89±0.71
Religious belief	Yes	50 (9.14)	2.73±0.27
	No	497 (90.86)	2.81±0.68
Complications	Yes	495 (90.49)	2.94±0.52
	No	52 (9.51)	1.51±0.28^**^
Dialysis modality	HD	130 (23.77)	2.78±0.58
	HD+HDF	89 (16.27)	2.85±0.52
	HD+HP	58 (10.60)	2.94±0.77
	HD+HDF+HP	270 (49.36)	2.77±0.69
Duration on dialysis, years	<1	55 (10.05)	2.68±0.77
	1-5	195 (35.65)	2.85±0.57
	≥5	297 (54.30)	2.79±0.67
Primary kidney disease	Primary glomerular disease	215 (39.30)	2.81±0.65
	Hypertension	176 (32.18)	2.83±0.66
	Diabetes mellitus	98 (17.92)	2.67±0.69
	Others	58 (10.60)	2.92±0.53

**P < 0.01; HD, hemodialysis; HDF, hemodiafiltration; HP, hemoperfusion.

### Correlations among perceived stress, sleep disturbance, and demoralization

As depicted in **Table 3**, the mean scores of the demoralization scales based on the cutoff point were high, and demoralization was significantly linked with perceived stress and sleep disturbance. Perceived stress was positively correlated with both sleep disturbance (r = 0.287, P < 0.01), and demoralization (r = 0.331, P < 0.01). Sleep disturbance was positively correlated with demoralization (r = 0.558, P < 0.01).

**Table 3:** The Pearson correlation among perceived stress, sleep disturbance, and demoralization.

Variable	Mean	SD	1	2	3
perceived stress	2.54	0.61	1		
sleep disturbance	9.35	1.97	0.287^**^	1	
demoralization	2.80	0.65	0.331^**^	0.558^**^	1

** *P* < 0.01.

### Regression analysis of sociodemographic and clinical characteristic, perceived stress, sleep disturbance, and demoralization

The HMR models of demoralization are illustrated in **Table 4**. Perceived stress was significantly positively correlated with demoralization, accounting for 10.2% of the variance. Sleep disturbance also showed a significantly positive correlation with demoralization, explaining an additional 21.7% of the variance. The regression coefficient (β) of perceived stress to demoralization was significantly reduced from 0.341 to 0.194 when sleep disturbance was added to the model. The results show that the potential effect of perceived stress on demoralization might be partially mediated by sleep disturbance.

**Table 4:** The hierarchical multiple regression models of demoralization.

Tools and measurements	Model 1		Model 2		Model 3	
	β	95% CI	β	95% CI	β	95% CI
Block 1 sociodemographic and clinical characteristics						
Employee status (yes vs. no)	0.160^**^	0.051~0.269	0.149^**^	0.045~0.253	0.110^*^	0.020~0.200
Monthly income						
(<5,000 Yuan vs. 5,000~10,000 Yuan)	0.097	-0.065~0.259	0.094	-0.059~0.247	0.105	-0.028~0.237
(<5,000 Yuan vs. ≥10,000 Yuan)	0.184	-0.158~0.526	0.213	-0.111~0.538	0.155	-0.125~0.436
Education level						
(junior school and below vs. senior high school)	-0.146	-0.315~0.022	-0.107	-0.268~0.053	-0.116	-0.255~0.023
(junior school and below vs. college and above)	-0.208	-0.602~0.185	-0.143	-0.516~0.230	-0.120	-0.443~0.203
Complications (yes vs. no)	0.325	-0.312~0.962	0.194	-0.410~0.798	0.212	-0.310~0.735
Block 2 perceived stress			0.341^**^	0.257~0.426	0.194^**^	0.118~0.270
Block 3 sleep disturbance					0.163^**^	0.139~0.187
R^2^	0.039		0.140		0.357	
Adjust R^2^	0.026		0.127		0.346	
△R^2^	0.039		0.102		0.217	

**P < 0.01, *P < 0.05.

### Mediating effect of sleep disturbance on the relationship between perceivedstress and demoralization

Model 4 in the SPSS PROCESS macro was used to address the mediating effect of sleep disturbance on the relationship between perceived stress and demoralization. In Step A, perceived stress was significantly associated with demoralization (β = 0.321, *p <*0.001). In Step B, perceived stress was significantly associated with sleep disturbance (β = 0.281, *p <*0.001). In Step C, both sleep disturbance and perceived stress were included in the mediation model and displayed a significant relationship with demoralization. Moreover, the standardized regression coefficient (β) of perceived stress to demoralization decreased from 0.321 to 0.182 (as shown in **Table 5**).

**Table 5:** Mediating effects of sleep disturbance on the relationship between perceived stress and demoralization.

Step	Path	B	SE	β	*t*	*p*
Step A	Perceived stress → Demoralization (c)	0.341	0.043	0.321	7.940	<0.001
Step B	Perceived stress → Sleep disturbance (a)	0.905	0.132	0.281	6.856	<0.001
Step C	Sleep disturbance → Demoralization (b)	0.163	0.012	0.493	13.469	<0.001
	Perceived stress → Demoralization (c’)	0.194	0.039	0.182	5.014	<0.001

B, unstandardized regression coefficient; SE, standard error; β, standardized regression coefficient.

**Figure 2:**
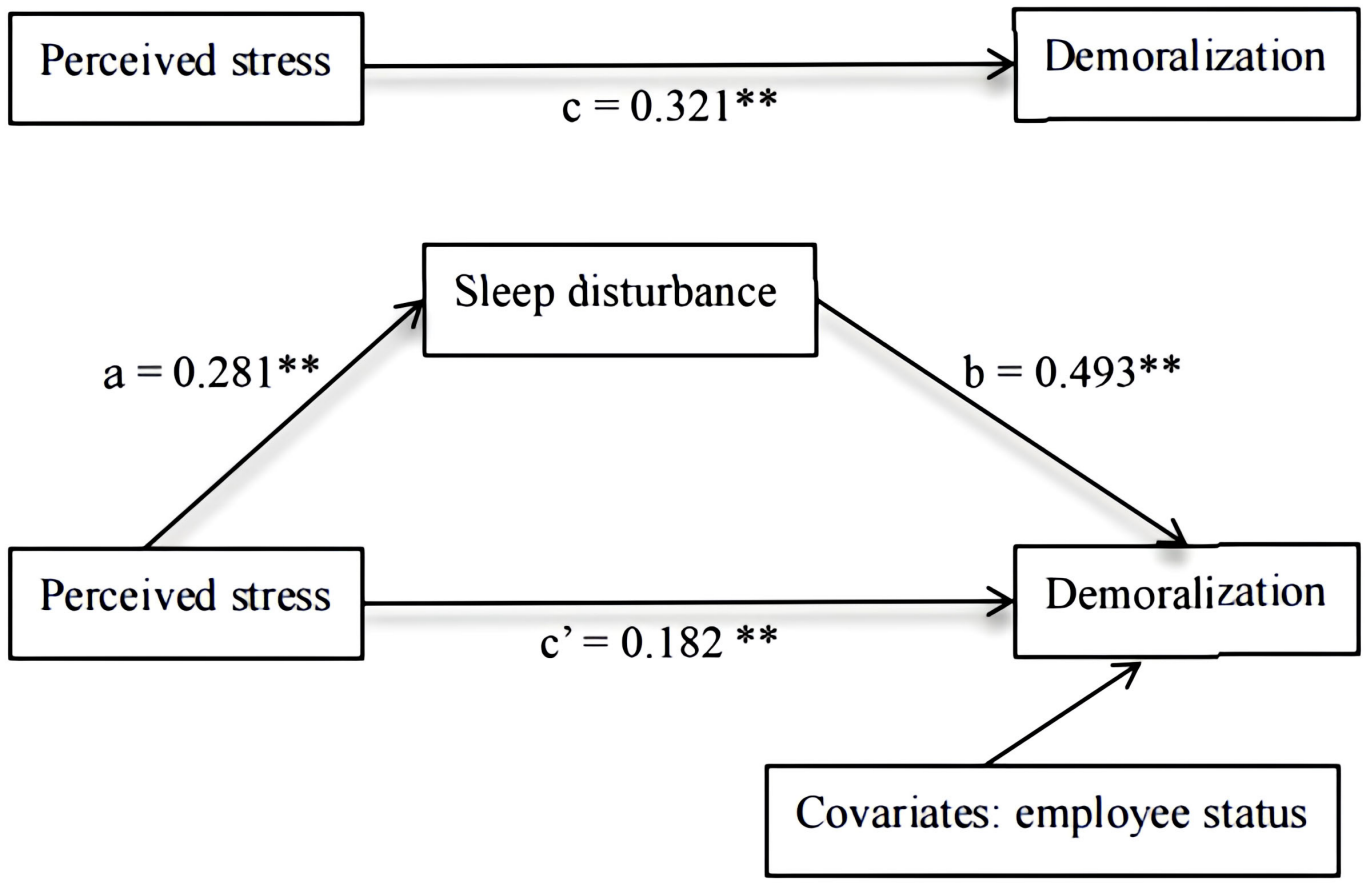
Final model (**P<0.01).

Furthermore, the significance of the indirect effect of perceived stress on demoralization through sleep disturbance (95% bootstrap CI = 0.1016, 0.2130) was confirmed by the nonparametric bootstrapping method. The indirect effect of perceived stress had an impact of 0.1543 produced by sleep disturbance as a mediator of demoralization. The indirect effect of sleep disturbance accounted for 43.75% of the total variance in demoralization influenced by perceived stress (as shown in **Table 6**). **Figure 2** shows the mediation model with the standardized path coefficients.

**Table 6:** Mediating model examination by bootstrap.

Effect	Perceived stress → Demoralization				Bootstrap 95% CI	Effect Ratio
	B	SE	*t*	*p*		
Total effect	0.353	0.043	8.183	<0.001	0.268-0.437	/
Direct effect	0.198	0.039	5.140	<0.001	0.122-0.274	56.25%
Indirect effect	0.154	0.029	/	/	0.102-0.213	43.75%

B, unstandardized regression coefficient; SE, standard error; CI, confidence interval.

## Discussion

This study found that hemodialysis patients had high levels of demoralization. The perceived stress of hemodialysis patients was positively associated with demoralization, and the mediating effect of sleep disturbance on the association between perceived stress and demoralization was found in the hemodialysis patients for the first time.

Among hemodialysis patients’ sociodemographic and clinical characteristics, employment status was the only significant predictor of demoralization. Hemodialysis patients with a job tended to have lower levels of demoralization than hemodialysis patients without a job, in line with previous reports (35, 36). For instance, Lee et al. (35) found unemployment to be associated with higher levels of demoralization among cancer outpatients. Being employed is associated with an individual’s economic security, social intercourse, self-realization, self-worth, power (control or dominance), and self-direction (independence in thought and action) (37), thereby protecting hemodialysis patients against demoralization by providing a sense of control and usefulness.

Perceived stress was positively associated with demoralization in hemodialysis patients, which is consistent with previous reports (11, 13). For example, Hsu et al. (11) found a higher level of stress to be associated with a higher level of demoralization. Perceived stress could be viewed as the sense of imbalance between the stressors experienced by an individual in daily life and his or her coping capability (38). Due to the nature of the disease and treatment, hemodialysis patients needing frequent hemodialysis sessions experience physical, emotional, and economic burdens (39). An average of six different chronic diseases, such as bone mineral disorders and hypertension, is expected for patients on hemodialysis (40). In addition to disease comorbidities and complications, patients with hemodialysis experience an average of eleven symptoms, such as anxiety, depression, fatigue, nausea, constipation, pain, and restless legs syndrome (40). As shown in **Table 2**, 83.91% of the patients did not have a job, and 56.67% of the patients had a family monthly income were less than 5,000 yuan, indicating that financial hardship for hemodialysis patients as a result of treatment costs and income loss is associated with decreased productivity (41). The physical, emotional, and economic burdens of hemodialysis patients can exceed an individual’s ability to cope, and this process could worsen the state of demoralization.

Sleep disturbance was positively and significantly related to demoralization in patients on hemodialysis. Sleep is a basic process of brain function recovery and is viewed as a fundamental aspect of physical and mental health (42). Healthy individuals with sleep deprivation experience a variety of negative health consequences, such as social withdrawal, changes in dietary patterns, interpersonal difficulties, fatigue, poor neurocognitive functioning, low positive emotion, high negative emotion, and emotion dysregulation (43–47). Some studies have also shown that sleep disturbance not only leads to cognitive and mental changes but also disrupts mental regulation and stability, such as experiencing restlessness and anxiety (48, 49). Under pressure, individuals with sleep disturbance have decreased immunity and weakened anti-infection and anti-inflammatory abilities, which could induce inflammatory reactions and lead to an increased number of disease comorbidities and complications (50). The above factors could lead to an increased sense of demoralization in hemodialysis patients.

This study not only further affirms the direct relationship between perceived stress and demoralization but is also the first to identify the mediating effect of sleep disturbance on the relationship between perceived stress and demoralization. Sleep disturbance strengthened the positive effect of perceived stress on demoralization. That is, the relationship between perceived stress and demoralization was enhanced because of sleep disturbance. Individuals with higher stress levels have higher demoralization levels when they encounter negative life events and usually experience sleep disturbance (51). Sleep disturbances are viewed as stress-related disorders and complications, such as nausea and pain (52). Patients with sleep disturbance view physical, emotional, and economic burdens as more stressful and use less effective coping strategies to cope with these burdens compared to people without sleep disturbance (53). These maladjusted cognitive and behavioral responses may conversely prevent an individual from falling asleep when they experience physical, emotional, and economic burdens, which could facilitate a worsened state of demoralization.

## Conclusion

Hemodialysis patients have high levels of demoralization. Our results confirm that perceived stress has a direct effect on demoralization and highlight the mediating effects of sleep disturbance on the relationship between perceived stress and demoralization, which has never been sufficiently studied in hemodialysis patients. The findings indicate that perceived stress is a key problem for patients on hemodialysis. The level of perceived stress for patients on hemodialysis should be evaluated and monitored appropriately. Psychological consultations should be provided to patients on hemodialysis when they perceive stress. As a precautionary measure, interventions focused on improving perceived stress with an emphasis on ameliorating sleep disturbances could be developed and provided to hemodialysis patients.

### Limitation

This study had several limitations. First, since perceived stress, sleep disturbance, and demoralization were measured using self-reported data, the relationships among the three variables might be vulnerable to response bias. Second, the results might not verify the causal relationship owing to the cross-sectional design. Future longitudinal studies are suggested to further verify the effects of perceived stress and sleep disturbance on demoralization and the mediating role of sleep disturbance. Third, The Pittsburgh Sleep Quality Index (PSQI), a useful measure of sleep quality, does not provide a diagnostic screening for specific sleep disorders such as insomnia. The Insomnia Severity Index (ISI) may offer a more specific assessment in this regard. Fourth, the study was only conducted in Yuxi, China, which limits the scope of application of the findings.

## Data Availability

The raw data supporting the conclusions of this article will be made available by the authors, without undue reservation.
